# O.3.1-2 Contribution of a set of physical activity types for adherence to the Dutch Physical Activity Guidelines

**DOI:** 10.1093/eurpub/ckad133.139

**Published:** 2023-09-11

**Authors:** Japke Kolkman, Marjolein Duijvestijn, Wanda Vos, Ellen de Hollander

**Affiliations:** National Institute for Public Health and the Environment (RIVM), The Netherlands; japke.kolkman@rivm.nl; National Institute for Public Health and the Environment (RIVM), The Netherlands; japke.kolkman@rivm.nl; National Institute for Public Health and the Environment (RIVM), The Netherlands; japke.kolkman@rivm.nl; National Institute for Public Health and the Environment (RIVM), The Netherlands; japke.kolkman@rivm.nl

## Abstract

**Purpose:**

To date, little is known about the contribution of different PA types to adherence to the PA guidelines. This study aims to identify which (combination of) PA types (e.g. sports, cycling during leisure time, gardening etc.) are the main contributors to adherence to the Dutch PA guidelines and whether this varies across population groups.

**Methods:**

Data of the Dutch Health Survey/Lifestyle monitor 2019 was used including 7616 respondents of 18 years and older. PA was assessed using the Short Questionnaire to Assess Health-enhancing physical activity (SQUASH). Prevalence rates of adherence to the PA guidelines were examined and the impact of excluding a particular PA type on these rates was assessed. Univariate and multiple logistic regression analyses were used to examine associations between demographic characteristics and adherence to the PA guidelines.

**Results:**

Prevalence rates decreased the most when either sports (12 percent points) or walking (17 percent points) or cycling (11 percent points) during leisure time was excluded in the total population and almost all population groups. Nevertheless, unadjusted- and adjusted odds ratios revealed that the importance of these PA types for adherence to the PA guidelines differed between population groups. For example, when all PA types were included, women had lower odds (0.89; 0.81-0.97) to adhere to the PA guidelines compared to men. When excluding sports, the odds increased towards 1.02 (0.92-1.12). This means that for men sports is a more important contributor to adherence to the PA guidelines than for women.

**Conclusions:**

Sports, walking- and cycling during leisure time contributed the most to adherence to the PA guidelines. Nevertheless, differences in the contribution of PA types between population groups exist. PA policies should target different population groups by stimulating the PA types that are main contributors for adherence to the PA guidelines, but also look for the barriers to participate in PA types that have a smaller contribution to adherence to the PA guidelines.

**Support/Funding Source:**

This research was funded by the Ministry of Health, Welfare and Sports of the Netherlands.

